# Free energy of a chemotactic model with nonlinear diffusion

**DOI:** 10.1038/s41598-017-09369-w

**Published:** 2017-08-21

**Authors:** Seung Ki Baek, Beom Jun Kim

**Affiliations:** 1 Department of Physics, Pukyong National University, Busan, 48513 Korea; 2 Department of Physics, Sungkyunkwan University, Suwon, 16419 Korea

## Abstract

The Patlak-Keller-Segel equation is a canonical model of chemotaxis to describe self-organized aggregation of organisms interacting with chemical signals. We investigate a variant of this model, assuming that the organisms exert effective pressure proportional to the number density. From the resulting set of partial differential equations, we derive a Lyapunov functional that can also be regarded as the free energy of this model, and minimize it with a Monte Carlo method to detect the condition for self-organized aggregation. Focusing on radially symmetric solutions on a two-dimensional disc, we find that the chemical interaction competes with diffusion so that aggregation occurs when the relative interaction strength exceeds a certain threshold. Based on the analysis of the free-energy landscape, we argue that the transition from a homogeneous state to aggregation is abrupt yet continuous.

## Introduction

Ants communicate with each other through the use of pheromones to adjust their collective behaviour^1–3^. This mechanism often leads to intriguing self-organized patterns. For example, their foraging path can be understood as solving a certain optimization problem in terms of time and energy costs^4–9^, and the shape of the path is predictable by Fermat’s principle of least time^10–12^. From a biological point of view, especially in the context of natural selection, it is highly plausible that an ant colony benefits from the ability of organizing a foraging path. It is also worth noting that the key ingredient is not an individual ant with little computational capacity, but the interaction in a group of such ants. It is thus regarded as an example of emergent phenomena^13^ and the term ‘swarm intelligence’ has been coined to describe this idea. Various computational techniques can be categorized as based on swarm intelligence (see, e.g., refs 14 and 15). From a physical point of view, ants provide a good example of active matter^16^, which can aggregate^17^ or circulate^6^ spontaneously and exhibit peculiar mechanical properties^18^.

The Patlak-Keller-Segel equation is a canonical starting point to study organisms that interact by means of chemical attractants^19, 20^. This model treats the density of organisms *ρ*(**r**, *t*) and the concentration of chemical attractants *c*(**r**, *t*) as continuous variables, where **r** denotes spatial coordinates and *t* means time, and describes the interplay between them. The Patlak-Keller-Segel equation has been extensively studied by mathematicians and a variety of review papers are available (see, e.g., refs 21 and 22). One of characteristic features of this model is that the organisms can form a dense aggregate, developing a *δ*-function peak within a finite time, when the space has dimensionality *d* > 1. Although such a ‘blow-up’ phenomenon provides an approximate description for biological aggregation, it is not entirely realistic that the whole population collapses to a single point. Researchers have suggested various mechanisms to regularize this singularity: To name a few, there are density-dependent chemotactic sensitivity^23–26^, nonlinear diffusion^27, 28^, logistic damping^29^, cross diffusion^30^, and shear flows^31^. One may also refer to a review by Hillen and Painter^32^ for many variations of the classical Patlak-Keller-Segel model. One may also refer to ref. 33 to see how it can be used to describe the organization of a foraging path.

This work adopts the idea of nonlinear diffusion^27, 28^ to take into account the finite volume of the organisms, and analyse its consequences. Let us write down the following set of equations:

1 $$\frac{\partial \rho }{\partial t}=\nabla \cdot (-{\chi }_{0}\rho \nabla c+{D}_{0}\rho \nabla \rho )$$

2 $$\frac{\partial c}{\partial t}={f}_{0}\rho +{\nu }_{0}{\nabla }^{2}c-{g}_{0}c,$$

where *χ*_0_, *D*_0_, *f*_0_, *ν*_0_, and *g*_0_ are positive constants. The terms on the right-hand side of Eq. (1) represent chemotactic movement and nonlinear diffusion, respectively. On the other hand, the three terms on the right-hand side of Eq. (2) mean generation, diffusion, and degradation, respectively. According to the original derivation^27^, the nonlinear diffusion term derives from $\rho \nabla h(\rho )$ with a pressure function *h*(*ρ*) due to crowding. If the pressure is expanded as a power series of density, as in the virial expansion, the choice of *h*(*ρ*) ∝ *ρ* corresponds to the lowest-order approximation, because the zeroth order clearly vanishes as *h*(*ρ* = 0) = 0. Some numerical observations have been reported in this case^28, 32^. Although *h*(*ρ*) is effective pressure to describe collective motion phenomenologically, it is interesting to note that an ant aggregate has an elastic modulus, which has units of pressure, as a linear function of *ρ*, until the ants are so densely packed that their legs are compressed^18^. Note that the classical Patlak-Keller-Segel equation is interpreted as $h(\rho )\sim \,\mathrm{ln}\,\rho$ from this viewpoint.

In this work, we show that the system described by Eqs (1) and (2) has a Lyapunov functional whose time derivative is smaller than or equal to zero all the time. It will also be called the free energy on the analogy with statistical mechanics. In general, a Lyapunov functional is a powerful tool in analysing a dynamical system, and its existence can be utilized to study properties of a fixed point beyond the local stability analysis^34^. After examining two stationary states, of which one is homogeneous and the other is not, we investigate the Lyapunov functional in the normal-mode coordinates to examine the transition between the homogeneous and inhomogeneous states, restricting ourselves to radially symmetric solutions. We will minimize the Lyapunov functional with a Monte Carlo method because it is computationally efficient in studying long-time behaviour of the system. We then briefly check if the Monte Carlo results are consistent with those from the direct numerical integration of the partial differential equations. After characterizing the transition based on the free-energy landscape, we conclude this work.

## Analysis

In this section, we begin with deriving the Lyapunov functional of Eqs (1) and (2). We are interested in homogeneous and inhomogeneous solutions and a transition between them. Of course, their stability can be studied in a standard way by adding small perturbation with the lowest nonzero mode, as will be demonstrated below. However, our main point is that the transition from the homogeneous distribution to aggregation can be analysed in detail by means of the Lyapunov functional, which contains the full spectrum of possible modes in this system.

### Lyapunov functional

Before proceeding, we have to specify the boundary conditions of our model. In analysing Eqs (1) and (2), we consider a two-dimensional disc of radius *l* and choose the Neumann boundary conditions,

3 $$\frac{\partial \rho }{\partial r}=\frac{\partial c}{\partial r}=0$$

at *r* = 0 and *r* = *l*, where *r* ≡ |**r**| is the distance from the origin of the disc. This condition means that the organisms cannot enter or escape from the system across the boundary, which is the experimental situation under consideration. In other words, Eq. (1) is derived from a continuity equation with current ${\bf{j}}=-{\chi }_{0}\rho \nabla c+{D}_{0}\rho \nabla \rho$, which implies that it conserves the total mass of the organisms:

4 $$M={\int }_{0}^{2\pi }\,{\int }_{0}^{l}\,\rho (r,\theta )r\,dr\,d\theta =\int \,\rho (r,\theta )dV,$$

where *θ* means the angle in the polar coordinates and *dV* is a volume element.

If we assume that the chemical attractant reaches a stationary state very quickly, so that the left-hand side of Eq. (2) can be taken to be approximately zero, we can solve the equation for *c* ^35^. Let us consider the entire two-dimensional space for simplicity. The formal solution is then given as

5 $$c({\bf{x}})=-\frac{{f}_{0}}{{\nu }_{0}}\int \,d{\bf{y}}{\mathscr{G}}({\bf{x}}-{\bf{y}})\,\rho ({\bf{y}}),$$

where ${\mathscr{G}}$ is the Green function obtained in terms of *K*_0_, the modified Bessel function of the second kind, as follows:

6 $${\mathscr{G}}({\bf{x}}-{\bf{y}})=-\frac{1}{2\pi }{K}_{0}(\kappa |{\bf{x}}-{\bf{y}}|)$$

with $\kappa \equiv \sqrt{{f}_{0}/{\nu }_{0}}$. Plugging this into Eq. (1), we find that

7 $$\frac{\partial \rho }{\partial t}=\nabla \cdot (\rho \nabla \frac{\delta  {\mathcal E} }{\delta \rho })$$

with

8 $$ {\mathcal E} \equiv \frac{{D}_{0}}{2}\int \,{\rho }^{2}({\bf{x}})d{\bf{x}}+\frac{{f}_{0}{\chi }_{0}}{2{\nu }_{0}}\iint \,\rho ({\bf{x}}){\mathscr{G}}({\bf{x}}-{\bf{y}})\rho ({\bf{y}})d{\bf{x}}d{\bf{y}}\mathrm{.}$$

Note that the first term is equivalent to the participation ratio in the localization problem^36^, and the second term can be interpreted as interaction energy between organisms at a distance. The participation ratio is minimized when *ρ* is distributed homogeneously, whereas the effective interaction potential, Eq. (6), make the organisms attract each other. If diffusion is dominant, i.e., ${D}_{0}{\nu }_{0}\gg {f}_{0}{\chi }_{0}$, the interaction term becomes negligible and the aggregation mediated by the chemical attractants will be suppressed. From Eqs (7) and (8), it is straightforward to see that

9 $$\frac{d {\mathcal E} }{dt}=-\int \,{|\nabla \frac{\delta  {\mathcal E} }{\delta \rho }|}^{2}\,\rho ({\bf{x}})d{\bf{x}},$$

which implies that ${\mathcal E}$ never increases as time goes by.

We have derived Eq. (8) under the restriction that ∂*c*/∂*t* = 0 only because ${\mathcal E}$ provides a simple physical interpretation in terms of *ρ* only. In fact, it is possible to construct a complete Lyapunov functional without such a restriction: Let us rescale the variables as *τ* = *D*_0_ *t* and $c^{\prime} =\frac{{\chi }_{0}}{{D}_{0}}c$. to rewrite Eqs (1) and (2) as

10 $$\frac{\partial \rho }{\partial \tau }=\nabla \cdot (-\rho \nabla c^{\prime} +\rho \nabla \rho )=\nabla \cdot (\rho \nabla Z)$$

11 $$\frac{{\chi }_{0}}{{\nu }_{0}}\frac{\partial c^{\prime} }{\partial \tau }={\nabla }^{2}c^{\prime} -\frac{{g}_{0}}{{\nu }_{0}}c^{\prime} +\frac{{f}_{0}{\chi }_{0}}{{D}_{0}{\nu }_{0}}\rho ,$$

where *Z* ≡ *ρ* − *c*′. We can show that

12 $$\int \,dV\,Z\frac{\partial \rho }{\partial \tau }=\int \,dV\,\nabla \cdot (Z\rho \nabla Z)-\int \,dV\,\rho {|\nabla Z|}^{2},$$

where the first term on the right-hand side vanishes due to the boundary conditions. By using Eq. (12), we can also show the following:

13 $$\frac{d}{d\tau }\int \,dV\,\rho Z=\int \,dV\,\rho \frac{\partial Z}{\partial \tau }+\int \,dV\,Z\frac{\partial \rho }{\partial \tau }$$

14 $$\quad \quad \quad \quad \,\,\,=\frac{d}{d\tau }\int \,dV\,\frac{{\rho }^{2}}{2}-\int \,dV\,\rho \frac{\partial c^{\prime} }{\partial \tau }-\int \,dV\,\rho {|\nabla Z|}^{2}\mathrm{.}$$

In addition, we have the following equality:

15 $$0=\int \,dV\,\nabla \cdot (\frac{\partial c^{\prime} }{\partial \tau }\nabla c^{\prime} )$$

16 $$\,\,\,=\frac{{\chi }_{0}}{{\nu }_{0}}\int \,dV\,{(\frac{\partial c^{\prime} }{\partial \tau })}^{2}+\frac{{g}_{0}}{{\nu }_{0}}\int \,dV\,c^{\prime} \frac{\partial c^{\prime} }{\partial \tau }-\frac{{f}_{0}{\chi }_{0}}{{D}_{0}{\nu }_{0}}\int \,dV\,\rho \frac{\partial c^{\prime} }{\partial \tau }+\frac{d}{d\tau }\int \,dV\,\frac{{|\nabla c^{\prime} |}^{2}}{2}\mathrm{.}$$

Plugging Eq. (14) into Eq. (16), we get

17 $$-\frac{dW}{d\tau }=\frac{{\chi }_{0}}{{\nu }_{0}}\int \,dV\,{(\frac{\partial c^{\prime} }{\partial \tau })}^{2}+\frac{{f}_{0}{\chi }_{0}}{{D}_{0}{\nu }_{0}}\int \,dV\,\rho {|\nabla Z|}^{2},$$

where

18 $$W\equiv \frac{{f}_{0}{\chi }_{0}}{{D}_{0}{\nu }_{0}}\int \,dV\,(\frac{1}{2}{\rho }^{2}-\rho c^{\prime} )+\frac{{g}_{0}}{2{\nu }_{0}}\int \,dV\,{|c^{\prime} |}^{2}+\int \,dV\,\frac{{|\nabla c^{\prime} |}^{2}}{2}\mathrm{.}$$

It is clear from Eq. (17) that *dW*/*dτ* cannot be positive so that *W* does not increase when the system evolves according to Eqs (1) and (2). For this reason, this quantity is sometimes called the *free energy* of this system. The time derivative *dW*/*dτ* equals zero if ∂*c*′/∂*τ* = 0 and ${\bf{j}}\propto \nabla Z=0$ everywhere that *ρ* > 0. The first integral of Eq. (18) consists of the participation ratio and the potential energy due to the coupling between *ρ* and *c*, whereas the other two integrals describe the chemical energy^37^. Likewise, one can argue that Eq. (17) contains the chemical production term ∝(∂*c*/∂*t*)^2^ on its right-hand side, and that the last term corresponds to something referred to as entropy production in the classical Patlak-Keller-Segel model because it is related to the time derivative of the Shannon entropy^37^. In our nonlinear-diffusion model, the last term of Eq. (17) may be regarded as generalized entropy production in terms of the Tsallis entropy^38^. It is also worth noting that the integrands in Eq. (18) are all quadratic, which will turn out to be useful for our analysis.

### Linear stability of a homogeneous stationary solution

Equations (1) and (2) admit a homogeneous stationary solution $\rho =\frac{{g}_{0}}{{f}_{0}}c={\rho }_{{\rm{const}}}$, where *ρ*_const_ = *M*/(*πl* ^2^) from Eq. (4). In this state, Eq. (18) yields

19 $$W=\frac{{M}^{2}}{2\pi {l}^{2}}\frac{{f}_{0}{\chi }_{0}}{{D}_{0}{\nu }_{0}}(1-\frac{{f}_{0}{\chi }_{0}}{{D}_{0}{g}_{0}})\mathrm{.}$$

The standard linear stability analysis assumes small perturbations *ε*_*ρ*_ and *ε*_*c*_ around this homogeneous solution to assume *ρ*(**r**, *t*) = *ρ*_const_ + *ε*_*ρ*_(**r**, *t*) and $c({\bf{r}},\,t)=\frac{{f}_{0}}{{g}_{0}}{\rho }_{{\rm{const}}}+{\varepsilon }_{c}({\bf{r}},\,t)$. By collecting linear terms in *ε*_*ρ*_ and *ε*_*c*_, we obtain

20 $$\frac{\partial }{\partial t}(\begin{array}{c}{\varepsilon }_{\rho }\\ {\varepsilon }_{c}\end{array})=(\begin{array}{cc}0 & 0\\ {f}_{0} & -{g}_{0}\end{array})\,(\begin{array}{c}{\varepsilon }_{\rho }\\ {\varepsilon }_{c}\end{array})+(\begin{array}{cc}{D}_{0}{\rho }_{{\rm{const}}} & -{\chi }_{0}{\rho }_{{\rm{const}}}\\ 0 & {\nu }_{0}\end{array})\,{\nabla }^{2}(\begin{array}{c}{\varepsilon }_{\rho }\\ {\varepsilon }_{c}\end{array})\mathrm{.}$$

Suppose that the perturbations are described as cylindrical harmonics, satisfying the following equation:

21 $$({\nabla }^{2}+{k}^{2})\,(\begin{array}{c}{\varepsilon }_{\rho }\\ {\varepsilon }_{c}\end{array})=0.$$

Each mode then takes the form of *J*_*n*_(*kr*)*e* ^±*inθ*^ *e* ^*ηt*^, where *J*_*n*_ means the Bessel function and *η* is its growth rate. The Neumann boundary conditions are expressed as $\frac{\partial }{\partial r}{J}_{n}(kl)=0$. The lowest mode is thus found at *n* = 0, which means radially symmetric density fluctuations concentrated around the origin. The first zero of *J*_1_ is located at *kl* ≈ 3.832…. If we solve the resulting eigenvalue problem:

22 $${\eta }^{2}+[{k}^{2}({D}_{0}{\rho }_{{\rm{const}}}+{\nu }_{0})+{g}_{0}]\eta +{\rho }_{{\rm{const}}}[{k}^{2}{D}_{0}({g}_{0}+{k}^{2}{\nu }_{0})-{k}^{2}{f}_{0}{\chi }_{0}]=\mathrm{0,}$$

the stability condition is obtained as *k* ^2^ *D*_0_(*g*_0_ + *k* ^2^ *ν*_0_) − *k* ^2^ *f*_0_ *χ*_0_ > 0. Note that it is independent of *ρ*_const_, differently from the classical Patlak-Keller-Segel model^39^, so that the system does not need critical mass for instability. This feature is, however, due to our particular choice of nonlinear diffusion. We find a necessary condition for the lowest mode to grow in time as follows:

23 $${k}^{2}{l}^{2}\approx 14.684 < (\frac{{f}_{0}{\chi }_{0}}{{D}_{0}{\nu }_{0}}-\frac{{g}_{0}}{{\nu }_{0}})\,{l}^{2}={K}^{2}{l}^{2},$$

where

24 $$K\equiv \sqrt{\frac{{f}_{0}{\chi }_{0}}{{D}_{0}{\nu }_{0}}-\frac{{g}_{0}}{{\nu }_{0}}}\mathrm{.}$$

If we assume that ${g}_{0}\ll 1$, the expression inside the square root of Eq. (24) is interpreted as a ratio between chemotactic strength and diffusivity. This small-*g*_0_ limit is often plausible without altering the essential physics, because some ant pheromones last for days^40^. Equation (23) suggests that *Kl* will be an important dimensionless parameter that governs the aggregation phenomenon.

In addition, if the disc is so large that the boundary effects are negligible and there is a continuous spectrum of possible wavenumbers, the initial stage of instability from the homogeneous solution is governed by the most unstable mode with *k* = *k*_*u*_ such that maximizes the positive *η* ^26^. The wavenumber *k*_*u*_ can be expressed by the following formula:

25 $${k}_{u}^{2}={f}_{0}{\chi }_{0}\sqrt{\frac{{\rho }_{{\rm{const}}}}{{D}_{0}{\nu }_{0}}}{(\frac{1}{\sqrt{{D}_{0}{\rho }_{{\rm{const}}}}+\sqrt{{\nu }_{0}}})}^{2},$$

where we take the limit of *g*_0_ → 0 to simplify the expression. Equation (25) will determine the typical length scale between aggregates, when the homogeneous initial state becomes unstable.

### Inhomogeneous stationary solution

Let us now consider a radially symmetric stationary aggregate. The boundary conditions make the flux vanish everywhere, i.e., ${\bf{j}}=-{\chi }_{0}\rho \nabla c+{D}_{0}\rho \nabla \rho =0$. It implies that

26 $$\rho =\frac{{\chi }_{0}}{{D}_{0}}(c-{c}_{0})$$

with a constant of integration *c*_0_. Substituting Eq. (26) into Eq. (2) with the stationarity condition, we obtain an inhomogeneous Helmholtz equation:

27 $$0=\frac{{f}_{0}{\chi }_{0}}{{D}_{0}}(c-{c}_{0})+{\nu }_{0}{\nabla }^{2}c-{g}_{0}c,$$

which has the following radially symmetric solution:

28 $$c(r)=A{J}_{0}(Kr)+\frac{{f}_{0}{\chi }_{0}}{{K}^{2}{D}_{0}{\nu }_{0}}{c}_{0},$$

where *A* is a constant describing the amplitude of aggregation, *J*_*n*_ is the Bessel function, and the wavenumber *K* has been defined in Eq. (24) above. Obviously, the solution is feasible only when the boundary condition is satisfied by ${\tfrac{d}{dr}{J}_{0}(Kr)|}_{r=l}=-K{J}_{1}(Kl)=0$, and let us suppose that this is the case. The constant *A* is bounded by a condition that both *ρ* and *c* must be non-negative everywhere. If we plug Eq. (28) into Eq. (26), we find that

29 $$\rho (r)=\frac{{\chi }_{0}}{{D}_{0}}[A{J}_{0}(Kr)+(\frac{{f}_{0}{\chi }_{0}}{{K}^{2}{D}_{0}{\nu }_{0}}-1){c}_{0}]\mathrm{.}$$

The unknown constant *c*_0_ can be explicitly determined from Eq. (4) because ${\int }_{0}^{l}\,{J}_{0}(Kr)r\,dr=0$ as long as the boundary conditions are satisfied. After some algebra, we can write the results as

30 $$\rho =\frac{{\chi }_{0}}{{D}_{0}}A{J}_{0}+{\rho }_{{\rm{const}}}$$

31 $$c=A{J}_{0}+{c}_{{\rm{const}}},$$

where *ρ*_const_ and *c*_const_ define the homogeneous solution. We substitute these results into Eq. (18) to calculate the Lyapunov functional:

32 $$\begin{array}{rcl}W & = & \frac{{f}_{0}{\chi }_{0}}{{D}_{0}{\nu }_{0}}\int \,dV[\frac{1}{2}{(\frac{{\chi }_{0}}{{D}_{0}}A{J}_{0}+m)}^{2}-(\frac{{\chi }_{0}}{{D}_{0}}A{J}_{0}+m)\frac{{\chi }_{0}}{{D}_{0}}(A{J}_{0}+\frac{{f}_{0}{\chi }_{0}}{{K}^{2}{D}_{0}{\nu }_{0}}{c}_{0})]\\  &  & +\frac{{g}_{0}}{2{\nu }_{0}}\int \,dV[\frac{{\chi }_{0}^{2}}{{D}_{0}^{2}}{(A{J}_{0}+\frac{{f}_{0}{\chi }_{0}}{{K}^{2}{D}_{0}{\nu }_{0}}{c}_{0})}^{2}]+\frac{1}{2}\int \,dV\frac{{\chi }_{0}^{2}}{{D}_{0}^{2}}{(KA{J}_{1})}^{2}\end{array}$$

33 $$\begin{array}{rcl} & = & -\frac{{f}_{0}{\chi }_{0}^{3}{A}^{2}}{2{D}_{0}^{3}{\nu }_{0}}\int \,dV{J}_{0}^{2}+(\frac{1}{2}\frac{{f}_{0}{\chi }_{0}}{{D}_{0}{\nu }_{0}}{m}^{2}-\frac{{f}_{0}^{2}{\chi }_{0}^{3}}{{K}^{2}{D}_{0}^{3}{\nu }_{0}^{2}}m{c}_{0})\pi {l}^{2}\\  &  & +\frac{{g}_{0}{\chi }_{0}^{2}{A}^{2}}{2{D}_{0}^{2}{\nu }_{0}}\int \,dV{J}_{0}^{2}+\frac{{f}_{0}^{2}{\chi }_{0}^{4}{g}_{0}}{2{K}^{4}{D}_{0}^{4}{\nu }_{0}^{3}}{c}_{0}^{2}\pi {l}^{2}+\frac{{\chi }_{0}^{2}{K}^{2}{A}^{2}}{2{D}_{0}^{2}}\int \,dV{J}_{1}^{2}\mathrm{.}\end{array}$$

We can see that the three integrals on the last line vanish altogether, if we note the definition of *K* [Eq. (24)] and the following identity:

34 $$\begin{array}{rcl}{K}^{2}{\int }_{0}^{l}\,r{J}_{0}^{2}(Kr)\,dr & = & K{\int }_{0}^{l}\,\frac{d}{dr}[r{J}_{1}(Kr)]\,{J}_{0}(Kr)\,dr\\  & = & -K{\int }_{0}^{l}\,r{J}_{1}(Kr)\,\frac{d}{dr}{J}_{0}(Kr)\,dr\\  & = & {K}^{2}{\int }_{0}^{l}\,r{J}_{1}{(Kr)}^{2}\,dr,\end{array}$$

which is valid under our assumption that *J*_1_(*Kl*) = 0. As a result, we obtain

35 $$W=\frac{{M}^{2}}{2\pi {l}^{2}}\frac{{f}_{0}{\chi }_{0}}{{D}_{0}{\nu }_{0}}(1-\frac{{f}_{0}{\chi }_{0}}{{g}_{0}{D}_{0}}),$$

which is identical to the Lyapunov functional of the homogeneous solution [Eq. (19)]. It is consistent with the fact that the solution with *K* has neutral stability in the linear-stability analysis [see, e.g., Eq. (23)], according to which the radially symmetric mode ∝*J*_0_(*kr*) can survive only when *k* is smaller than *K*. Although we have assumed that the wavenumber *K* is compatible with the boundary condition, it is actually independent of *l*, which implies that the stationarity condition cannot be met exactly. If a perturbative mode with *k* < *K* appears from the homogeneous state with satisfying the boundary conditions, therefore, it cannot be stationary: Its amplitude will grow exponentially at first, but cannot become arbitrarily large because of the non-negativity of *ρ* and *c*. The growth will stop when *A* reaches the largest value that does not violate the non-negativity. This scenario seems to suggest a jump in *A* as *K* crosses a threshold, and this scenario will be scrutinized below by considering a full spectrum of normal modes.

### Normal-mode expansion

Let us decompose *ρ* and *c* into normal modes:

36 $$\rho (r,\theta ,t)={\rho }_{{\rm{const}}}+\sum _{p=0}^{\infty }\,\sum _{m=1}^{\infty }\,{J}_{p}({j}_{p,m}^{^{\prime} }r/l)\,[{E}_{pm}(t)\,\cos \,p\theta +{F}_{pm}(t)\,\sin \,p\theta ]$$

37 $$c(r,\theta ,t)={c}_{{\rm{const}}}+\sum _{p=0}^{\infty }\,\sum _{m=1}^{\infty }\,{J}_{p}({j}_{p,m}^{^{\prime} }r/l)\,[{G}_{pm}(t)\,\cos \,p\theta +{H}_{pm}(t)\,\sin \,p\theta ],$$

where ${j}_{pm}^{^{\prime} }$ denotes the *m*th zero of $\tfrac{d}{dx}{J}_{p}(x)$. Note that

38 $${\int }_{0}^{2\pi }\,{\int }_{0}^{l}\,r{J}_{p}({j}_{pm}^{^{\prime} }r/l)\,{e}^{ip\theta }\,dr\,d\theta =\mathrm{0,}$$

so that Eq. (36) automatically conserves the total mass $M={\int }_{0}^{2\pi }\,{\int }_{0}^{l}\,\rho (r,\theta )r\,dr\,d\theta ={\rho }_{{\rm{const}}}\pi {l}^{2}$. Likewise, the total amount of the chemical attractant is given as *c*_const_ *πl* ^2^, which is, however, a function of time in general. It is straightforward to see the following orthogonality relation

39 $${\int }_{0}^{l}\,r{J}_{p}({j}_{pu}^{^{\prime} }r/l)\,{J}_{p}({j}_{pw}^{^{\prime} }r/l)\,dr=-\frac{{l}^{2}}{2}{J}_{p}({j}_{pu}^{^{\prime} })\,\frac{{d}^{2}}{d{x}^{2}}{J}_{p}({j}_{pu}^{^{\prime} })\,{\delta }_{uw}=-\frac{{l}^{2}}{2}{\varphi }_{pu}{\delta }_{uw},$$

where *δ*_*uw*_ is the Kronecker delta and ${\varphi }_{pu}\equiv {J}_{p}({j}_{pu}^{^{\prime} })\,\tfrac{{d}^{2}}{d{x}^{2}}{J}_{p}({j}_{pu}^{^{\prime} })$.

We will rewrite the Lyapunov functional [Eq. (18)] by using Eqs (36) and (37)]. The first term needs an integral of *ρ* ^2^ over the disc, which can be expressed as

40 $$\frac{1}{\pi {l}^{2}}\int \,{\rho }^{2}dV={\rho }_{{\rm{const}}}^{2}-\sum _{m=1}^{\infty }\,[{\varphi }_{0m}{E}_{0m}^{2}+\frac{1}{2}\sum _{p=1}^{\infty }\,{\varphi }_{pm}\,({E}_{pm}^{2}+{F}_{pm}^{2})]$$

by using the orthogonality relations. The integrals of *ρc* and *c* ^2^ can be done in a similar way. However, the last part of the Lyapunov functional [Eq. (18)] is more complicated: It is involved with an integral of ${|\nabla c|}^{2}$, which is decomposed into two terms:

41 $$\int \,{|\nabla c|}^{2}dV={\int }_{0}^{2\pi }\,{\int }_{0}^{l}\,{|\frac{\partial c}{\partial r}|}^{2}r\,dr\,d\theta +{\int }_{0}^{2\pi }\,{\int }_{0}^{l}\,\frac{1}{{r}^{2}}{|\frac{\partial c}{\partial \theta }|}^{2}r\,dr\,d\theta .$$

We again substitute Eqs (36) and (37) here to obtain

42 $$\begin{array}{rcl}{\int }_{0}^{2\pi }\,{\int }_{0}^{l}\,{|\frac{\partial c}{\partial r}|}^{2}r\,dr\,d\theta  & = & \pi \sum _{m=1}^{\infty }\,{j}_{1m}^{2}{J}_{0}^{2}({j}_{1m}){G}_{0m}^{2}\\  &  & +\,\frac{\pi }{{l}^{2}}\sum _{p=1}^{\infty }\,\sum _{mn}\,{j}_{pm}^{^{\prime} }{j}_{pn}^{^{\prime} }({G}_{pm}{G}_{pn}+{H}_{pm}{H}_{pn})\\  &  & \times {\int }_{0}^{l}\,r({\tfrac{d{J}_{p}(x)}{dx}|}_{x={j}_{pm}^{^{\prime} }r/l})\,({\tfrac{d{J}_{p}(x)}{dx}|}_{x={j}_{pn}^{^{\prime} }r/l})\,dr\end{array}$$

and

43 $$\begin{array}{rcl}{\int }_{0}^{2\pi }\,{\int }_{0}^{l}\,\frac{1}{{r}^{2}}{|\frac{\partial c}{\partial \theta }|}^{2}r\,dr\,d\theta  & = & \pi \sum _{p=1}^{\infty }\,\sum _{mn}\,{p}^{2}({G}_{pm}{G}_{pn}+{H}_{pm}{H}_{pn})\\  &  & \times {\int }_{0}^{l}\,\frac{1}{r}{J}_{p}({j}_{pm}^{^{\prime} }r/l)\,{J}_{p}({j}_{pn}^{^{\prime} }r/l)\,dr\mathrm{.}\end{array}$$

Note that the results still have the triple sums over *p*, *m*, and *n*, because we cannot enjoy the orthogonality between *m* and *n* when performing the integrals over *r*.

**Figure 1:**
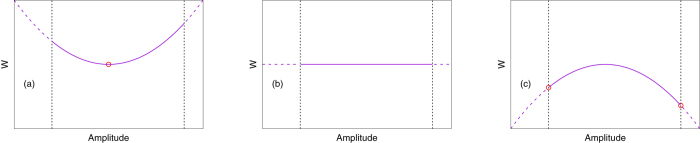
Sketches of the Lyapunov functional *W* along a principal axis, a combination of the amplitudes *E*_01_ and *G*_01_, when (**a**) *Kl* < *j*_11_, (**b**) *Kl* = *j*_11_, and (**c**) *Kl* > *j*_11_, respectively. The vertical dotted lines represent the physical constraint that both *ρ* and *c* should be non-negative, so that the system can explore only the landscapes of *W* drawn with solid lines. The small red circles show local minima of the given landscapes.

To circumvent the time-consuming evaluation of the triple sums, we focus on radially symmetric solutions by setting *p* = 0. If *j*_*pm*_ denotes the *m*th zero of *J*_*p*_(*x*), we can identify ${j}_{0m}^{^{\prime} }$ with *j*_1*m*_ because $\tfrac{d}{dx}{J}_{0}(x)=-{J}_{1}(x)$. Therefore, Eq. (39) further simplifies to

44 $${\int }_{0}^{l}\,r{J}_{0}({j}_{1u}r/l)\,{J}_{0}({j}_{1w}r/l)\,dr={\int }_{0}^{l}\,r{J}_{1}({j}_{1u}r/l)\,{J}_{1}({j}_{1w}r/l)\,dr=\frac{{l}^{2}}{2}{J}_{0}^{2}({j}_{1u})\,{\delta }_{uw},$$

where the first equality is derived in the same way as in Eq. (34), and the second one is the conventional orthogonality of the Bessel function^41^. Plugging Eqs (36) and (37) with *p* = 0 into the Lyapunov functional [Eq. (18)] and using the orthogonality, we find that

45 $$\begin{array}{rcl}\frac{W}{\pi {l}^{2}} & = & \frac{{f}_{0}{\chi }_{0}}{{D}_{0}{\nu }_{0}}[\frac{1}{2}({\rho }_{{\rm{const}}}^{2}+\sum _{m\mathrm{=1}}^{\infty }\,{J}_{0}^{2}({j}_{1m}){E}_{0m}^{2})\\  &  & -\frac{{\chi }_{0}}{{D}_{0}}({\rho }_{{\rm{const}}}{c}_{{\rm{const}}}+\sum _{m\mathrm{=1}}^{\infty }\,{J}_{0}^{2}({j}_{1m}){E}_{0m}{G}_{0m})]\\  &  & +\,\frac{{g}_{0}{\chi }_{0}^{2}}{2{D}_{0}^{2}{\nu }_{0}}({c}_{{\rm{const}}}^{2}+\sum _{m\mathrm{=1}}^{\infty }\,{J}_{0}^{2}({j}_{1m}){G}_{0m}^{2})\\  &  & +\frac{{\chi }_{0}^{2}}{2{D}_{0}^{2}{l}^{2}}\sum _{m\mathrm{=1}}^{\infty }\,{j}_{1m}^{2}{J}_{0}^{2}({j}_{1m}){G}_{0m}^{2}\end{array}$$

46 $$\begin{array}{rcl} & = & \sum _{m=0}^{\infty }\,\frac{1}{2}{J}_{0}^{2}({j}_{1m})(\begin{array}{cc}{E}_{0m} & \frac{{\chi }_{0}}{{D}_{0}}{G}_{0m}\end{array})\,(\begin{array}{cc}\frac{{f}_{0}{\chi }_{0}}{{D}_{0}{\nu }_{0}} & -\,\frac{{f}_{0}{\chi }_{0}}{{D}_{0}{\nu }_{0}}\\ -\,\frac{{f}_{0}{\chi }_{0}}{{D}_{0}{\nu }_{0}} & \frac{{g}_{0}}{{\nu }_{0}}+\frac{{j}_{1m}^{2}}{{l}^{2}}\end{array})\,(\begin{array}{c}{E}_{0m}\\ \frac{{\chi }_{0}}{{D}_{0}}{G}_{0m}\end{array}),\end{array}$$

where we have defined *E*_00_ ≡ *ρ*_const_, *G*_00_ ≡ *c*_const_, and *j*_10_ ≡ 0. We are interested in the minimum of Eq. (46), expecting that it captures the long-term behaviour of the system. The set of variables {*c*_const_, *E*_01_, *E*_02_, …, *G*_01_, *G*_02_, …} resulting from the minimization will be independent of the overall rescaling of *W* and thus determined by three dimensionless ratios, *χ*_0_/*D*_0_, *g*_0_/*f*_0_, and *ν*_0_/(*f*_0_ *l* ^2^). The first ratio measures the chemical sensitivity of the organism with respect to its nonlinear diffusivity. The next one measures the relative time scale between the generation and decay of the chemical attractant. Finally, the last one gives the typical time scale for the chemical attractant to diffuse into the whole system, measured with respect to the generation rate. Let us assume that each summand can be considered *separately* in this minimization problem. Then, for *m* = 0, only *c*_const_ varies, because *ρ*_const_ is fixed by the total mass *M*, and the optimal value for *c*_const_ equals (*f*_0_/*g*_0_)*ρ*_const_ as we have already seen in the homogeneous stationary solution. For every other *m* > 1, we have a simple quadratic function of *E*_0*m*_ and *G*_0*m*_. From an eigenvalue analysis, it is straightforward to see that the functional shape is elliptic when *j*_1*m*_ > *Kl* and hyperbolic otherwise, where *K* is defined by Eq. (24). In the former case, the minimum is located at *E*_0*m*_ = *G*_0*m*_ = 0. In the latter case, the minima of Eq. (46) are found at *E*_0*m*_ ∝ *G*_0*m*_ = ±∞, and the divergence must be regulated by the condition that both *ρ* and *c* are non-negative everywhere. The idea is sketched in Fig. 1 for *m* = 1. According to this argument, if *Kl* lies between *j*_11_ and *j*_12_, for example, we will observe two local minima, one for *E*_01_ ∝ *G*_01_ > 0 and the other for *E*_01_ ∝ *G*_01_ < 0, while all the other *E*_0*m*_’s and *G*_0*m*_’s with *m* > 1 remain suppressed to zero. An interesting point in this picture is that the Lyapunov functional becomes independent of the amplitude of aggregation if *Kl* exactly equals *j*_11_: An infinite number of states would have the same value of the Lyapunov functional. Therefore, even if the system converges to two different states as $Kl\to {j}_{11}^{+}$ and $Kl\to {j}_{11}^{-}$, respectively, there would be a continuous spectrum of states between them at *Kl* = *j*_11_.

## Numerical Results

**Figure 2:**
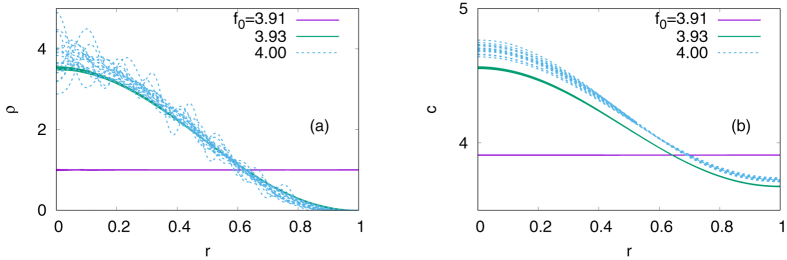
(**a**) Density of the organisms *ρ* and (**b**) the density of their chemical attractants *c*, obtained by minimizing a partial sum of Eq. (46) up to *m* = 19 with the Metropolis algorithm. We choose *χ*_0_ = 4, *ρ*_const_ = 1, *D*_0_ = 1, *ν*_0_ = 1, *g*_0_ = 1, and *l* = 1. For each *f*_0_, we run 20 independent samples, slowly lowering the ‘temperature’ from *T* = 10 to *T* = 0.

Let us choose *χ*_0_ = 4 and set other parameters, *ρ*_const_, *D*_0_, *ν*_0_, *g*_0_, and *l*, to unity. With these parameters, the system reaches the threshold for aggregation, *Kl* = *j*_11_, when ${f}_{0}={f}_{0}^{\ast }\approx 3.92$. We minimize the Lyapunov function for radially symmetric cases [Eq. (46)] with different values of *f*_0_ by means of the Metropolis algorithm (see Method for details). In evaluating Eq. (46) numerically, we have to replace the infinite series by a partial sum, and the spatial resolution of the resulting expression will be enhanced as we include more and more modes in the summation. Here, let us use a partial sum up to *m* = 19 because it already captures the overall behaviour correctly. This choice implies that we have to work with 39 variables of *c*_const_, *E*_01_, …, *G*_0*m*_. For the algorithm to search for the parameter space efficiently, we introduce a ‘temperature’ variable *T*, which helps the system escape from metastable local minima. We start with a sufficiently high temperature, say, *T* = 10^1^, to explore a wide region of the parameter space and then gradually lower the temperature down to *T* = 0. As argued above, we observe a sharp transition from a homogeneous solution to aggregation when *f*_0_ exceeds ${f}_{0}^{\ast }\approx 3.92$, and the aggregation pattern is approximated to *J*_0_(*j*_11_ *r*/*l*) [Fig. 2]. From *f*_0_ = 3.93 to *f*_0_ = 4.00, on the other hand, the system remains qualitatively the same, although small variations exist from sample to sample. To sum up, the behaviour at *Kl* ≈ *j*_11_ is indeed explained by the assumption that the minimization of Eq. (46) can be carried out term by term.

**Figure 3:**
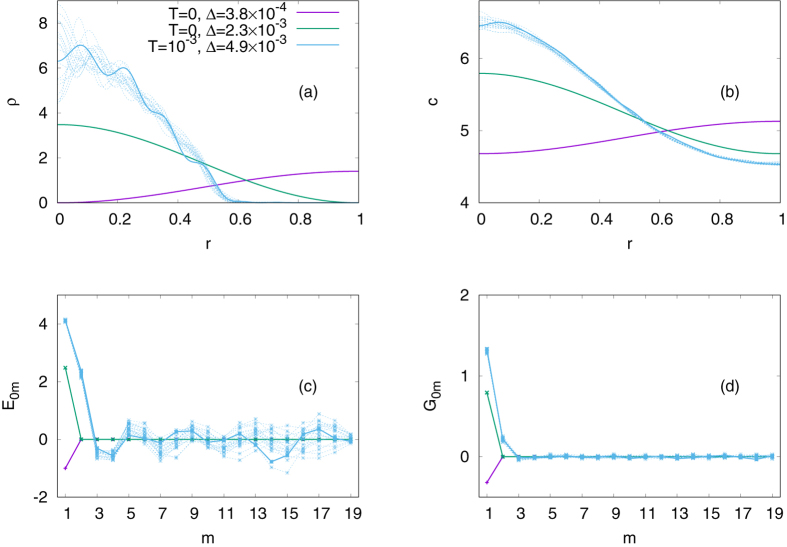
Minimization results of a partial sum of Eq. (46) up to *m* = 19, obtained by the Metropolis algorithm. In this plot, we show (**a**) the density of the organisms *ρ*, (**b**) that of the chemical attractants *c*, (**c**) the amplitudes *E*_0*m*_’s for describing *ρ*, and (**d**) *G*_0*m*_’s for *c*. We choose *f*_0_ = 5, *χ*_0_ = 4, *ρ*_const_ = 1, *D*_0_ = 1, *ν*_0_ = 1, *g*_0_ = 1, and *l* = 1. The initial condition is given by *c*_const_ = (*f*_0_/*g*_0_)*ρ*_const_ and *E*_0*m*_ = *G*_0*m*_ = 0 in each case. For the zero-temperature case, i.e., *T* = 0, the system approaches either of two different local minima, represented by the purple and green lines, respectively. If we instead slowly cool down the system from *T* = 10^1^ to *T* ≈ 10^−3^, we find high concentrations of *ρ* and *c* around *r* = 0 for all the 20 samples shown in this plot (the blue lines). Among the blue lines, the solid ones represent the sample with the best minimization result.

As *f*_0_ increases, however, the assumption loses validity. In Fig. 3, we plot our numerical minimization results with *f*_0_ = 5 while all the other parameters are the same as above. Then, the value of *Kl* ≈ 4.3589 still falls between *j*_11_ ≈ 3.8317 and *j*_12_ ≈ 7.0156. If *W*_ref_ denotes the value of the Lyapunov functional of the homogeneous solution, we see from Eq. (19) that *W*_ref_/*πl* ^2^ = −190. To see the minimization performance, we check a relative difference from this value,

47 $${\rm{\Delta }}\equiv \frac{{W}_{{\rm{ref}}}-W}{{W}_{{\rm{ref}}}}\mathrm{.}$$

**Figure 4:**
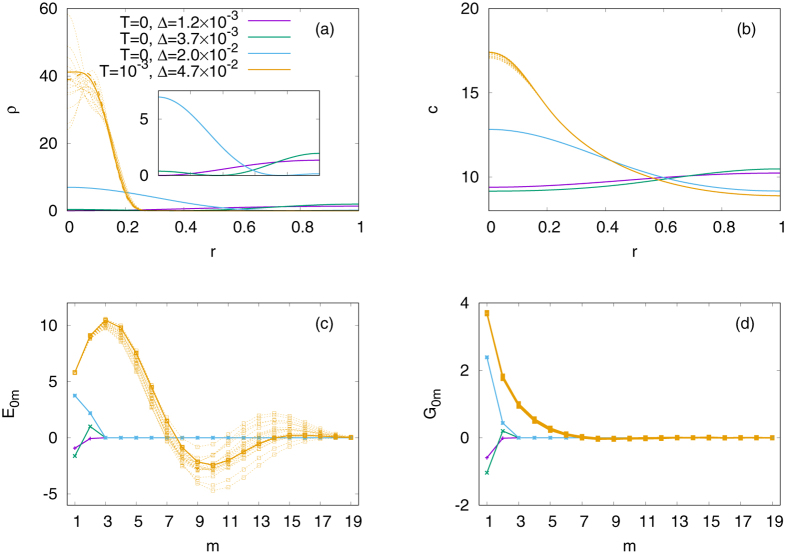
Minimization results of a partial sum of Eq. (46) up to *m* = 19, obtained by the Metropolis algorithm. We choose *f*_0_ = 10 and *χ*_0_ = 8, and keep all the others the same as in Fig. 3. (**a**) The density of the organisms. (Inset) If we run the zero-temperature Metropolis algorithm starting with ${E}_{01}={E}_{02}=\cdots ={G}_{01}={G}_{02}=\cdots =0$, the system approaches either of three different local minima, which are represented by the purple, green, and blue lines, respectively. We can also start from *T* = 10 and then cool down the system slowly. Performing this process with 20 independent samples, we plot their *ρ* at *T* = 10^−3^ with the orange lines. Among the orange lines, the solid ones represent the sample with the best minimization result. The other panels show (**b**) the density of the chemical attractants, (**c**) the normal-mode amplitudes for *ρ*, and (**d**) those for *c*, respectively.

We first run the Metropolis algorithm from *E*_0*m*_ = *G*_0*m*_ = 0 with fixing the temperature *T* to zero. We then find two different minima as expected: One describes a population concentration around *r* = 0, and the other shows an annular structure which is reminiscent of an ant mill^6^. These patterns nicely match with our picture in Fig. 1(c). Especially, the concentration around *r* = 0 is essentially the same pattern that we have shown in Fig. 2. However, if we start from *T* = 10^1^ and gradually lower the temperature down to *T* ≈ 10^−3^, a better minimization result is achieved and it is characterized by systematic deviations of *E*_0*m*_ from zero for $m\mathop{ < }\limits_{ \tilde {}}10$. The small yet finite temperature *T* ≈ 10^−3^ shows us how the modes are affected by environmental noises. Due to the excitation of high-*m* modes, we observe higher concentrations of *ρ* and *c* around the origin than expected from the zero-temperature case. Such coupling between modes would not be observed if Eq. (46) was minimized term by term. In Fig. 3, we see that *E*_01_ is considerably greater than that of the zero-temperature result. Higher modes with *m* > 1 should thus be excited to ensure the non-negativity of *ρ*, increasing *W*. Nevertheless, the reduction of *W* from *m* = 1 may well overtake the increment from *m* > 1, because each mode appears with a different weight in Eq. (46). The excitation of high-*m* modes becomes more pronounced as we go far above *j*_11_: For example, let us choose *f*_0_ = 10.0 and *χ*_0_ = 8.0, for which *Kl* ≈ 8.8882 is greater than *j*_12_ ≈ 7.0156 but lies below *j*_13_ ≈ 10.1735. We observe that the zero-temperature Metropolis algorithm ends up with one of three different minima shown in Fig. 4(a). Once again, the annealing procedure from *T* = 10^1^ to *T* = 10^−3^ finds a much better result, concentrating the most of the population around *r* = 0. Note that the amplitudes *E*_0*m*_ exhibit a nontrivial structure in Fig. 4(c). It actually extends to even higher *m* > 19 if we take more modes into account in computing Eq. (46), but those higher modes hardly affect the radius of the aggregate in Fig. 4(a).

When the distribution *ρ*(*r*) is given, the degree of aggregation can be estimated by the Shannon entropy:

48 $$S=-{\int }_{0}^{2\pi }\,{\int }_{0}^{l}\,\rho (r)\,\mathrm{log}\,\frac{\rho (r)}{{\rho }_{{\rm{const}}}}\,r\,dr\,d\theta .$$

**Figure 5:**
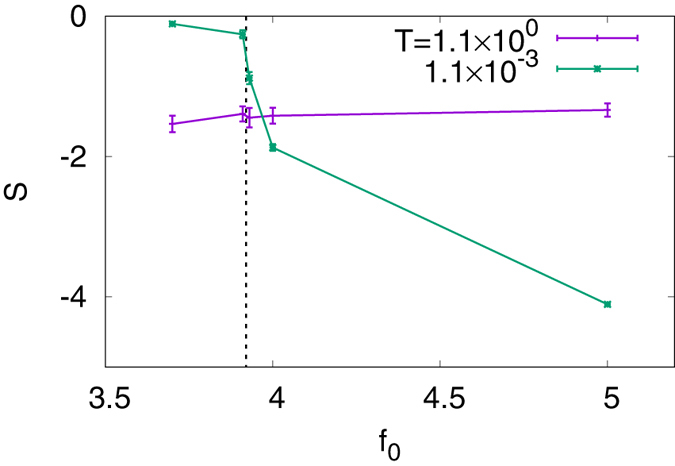
Shannon entropy [Eq. (48)] as a function of *f*_0_. The other parameters are the same as in Figs 2 and 3. For each data point, we take an average over 20 independent samples. The vertical dotted line represents ${f}_{0}={f}_{0}^{\ast }\approx 3.92$ to make *Kl* = *j*_11_.

Figure 5 shows *S* as a function of *f*_0_ at two different temperatures of the Monte Carlo calculation. The other parameters are set to the same as in Figs 2 and 3. When *T* is high, the system is insensitive to *f*_0_, and *S* does not show any significant change. For low *T*, on the other hand, it becomes clear that a jump of *S* exists in the vicinity of ${f}_{0}={f}_{0}^{\ast }$. Recall that the separability assumption predicts that the system undergoes stepwise changes as *f*_0_ increases, because *Kl* has to exceed *j*_1*m*_ to excite the *m*th mode (*m* = 1, 2, …). That is, if the assumption was valid everywhere, all the higher modes would remain suppressed unless *Kl* > *j*_12_, which requires *f*_0_ > 12.55. However, our Monte Carlo results have shown that modes tend to be coupled to each other to reduce the free energy to a greater extent than predicted by the separability assumption. In other words, it implies that *S* jumps only once at ${f}_{0}^{\ast }$ and then changes continuously for higher *f*_0_, which is indeed the case in Fig. 5.

It is also instructive to directly consider dynamics of Eqs (1) and (2) for the following reason: The idea behind our Monte Carlo calculation is that the result can describe long-time behaviour of the real dynamics. As mentioned in Method, the algorithm checks the non-negativity of *ρ* and *c* as well as the change of *W*, so that a Monte Carlo move will be rejected if it violates the non-negativity, even if it decreases *W*. On the other hand, the dynamics of Eqs (1) and (2) does not have such rejection but only continues with *dW*/*dt* ≤ 0 [Eq. (17)]. Therefore, one may well ask if the dynamics always confines the system in a physical region where both *ρ* and *c* are non-negative. Fortunately, the answer is yes, as has been proved in ref. 27. We can thus safely move on to the next question, i.e., whether the long-time behaviour is consistent with the Monte Carlo result. Under radial symmetry, the equations are written as

49 $$\frac{\partial \rho }{\partial t}=-{\chi }_{0}(\frac{\rho }{r}\frac{\partial c}{\partial r}+\frac{\partial \rho }{\partial r}\frac{\partial c}{\partial r}+\rho \frac{{\partial }^{2}c}{\partial {r}^{2}})+{D}_{0}[\frac{\rho }{r}\frac{\partial \rho }{\partial r}+{(\frac{\partial \rho }{\partial r})}^{2}+\rho \frac{{\partial }^{2}\rho }{\partial {r}^{2}}]$$

50 $$\frac{\partial c}{\partial t}={f}_{0}\rho +{\nu }_{0}(\frac{1}{r}\frac{\partial c}{\partial r}+\frac{{\partial }^{2}c}{\partial {r}^{2}})-{g}_{0}c.$$

**Figure 6:**
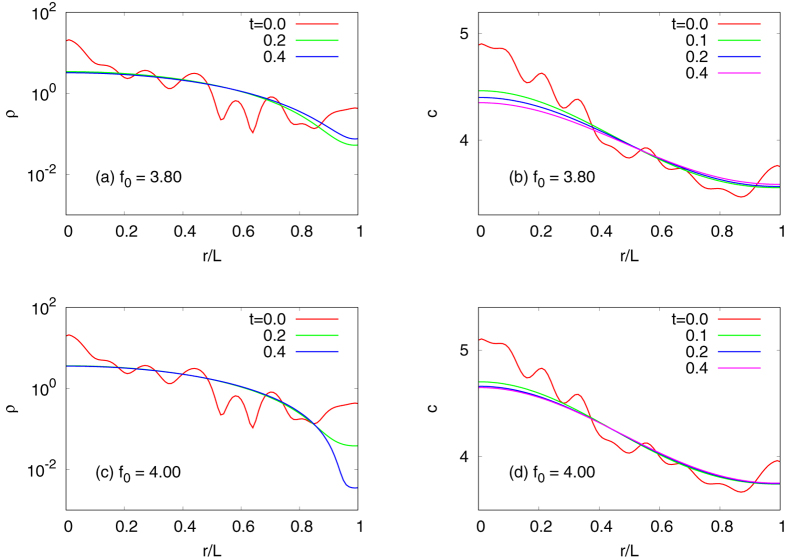
Direct numerical simulation of Eqs (1) and (2) under radial symmetry with the Forward-Time-Central-Space (FTCS) scheme. Panels (a,c) show *ρ*(*r*, *t*) and the others do *c*(*r*, *t*). We use the same parameter as in Figs 2 and 3, which means that the threshold corresponds to ${f}_{0}^{\ast }\approx 3.92$. Both for ${f}_{0}=3.80 < {f}_{0}^{\ast }$ (the upper panels) and ${f}_{0}=4.00 > {f}_{0}^{\ast }$ (the lower ones), the system starts from an identical configuration which is found by the Monte Carlo calculation at some high *T*. The only difference in the initial conditions is the total amount of the chemical attractants because we have set *c*_const_ = (*f*_0_/*g*_0_)*ρ*_const_ ∝ *f*_0_. The time step for integration is chosen to be Δ*t* = 10^−7^, and the horizontal axis is divided into 200 grid points. Note that the vertical axes are drawn on the log scale in panels (a,c) to see the behaviour of *ρ*(*r*, *t*) near the boundary at *r* = *L*.

We can integrate these equations numerically, e.g., with the Forward-Time Central-Space (FTCS) method^42^, and the results are given in Fig. 6. We still use the same parameters as in Figs 2 and 3 to have a threshold at ${f}_{0}={f}_{0}^{\ast }\approx 3.92$. As expected, both *ρ* and *c* become flatter as time goes by when ${f}_{0} < {f}_{0}^{\ast }$ [Fig. 6(a,b)]. On the other hand, when ${f}_{0} > {f}_{0}^{\ast }$ [Fig. 6(c,d)], *ρ* and *c* instead converge to inhomogeneous distribution functions, respectively, which exactly match with the ones in Fig. 2. Moreover, the time evolution undergoes critical slowing down as we approach ${f}_{0}^{\ast }$. It is consistent with the linear-stability analysis in which the eigenvalue governing the mode growth (or decay) vanishes at the threshold. However, we also note that the naive FTCS scheme becomes unstable at large *t*, violating the non-negativity condition. This must be a numerical artefact because, as mentioned above, the dynamics itself preserves the non-negativity of *ρ* and *c* ^27^. A better alternative could be to utilise the operator-splitting scheme^43^, incorporating exact solutions of the porous-medium equation (see, e.g., ref. 44).

## Summary

In summary, we have investigated a variant of the Patlak-Keller-Segel model in which pressure is assumed to increase linearly with the density of the organisms [Eqs (1) and (2)]. We have derived its Lyapunov functional *W* in Eq. (18), which may also be called the free energy of this system. The linear stability analysis of the homogeneous solution predicts a jump in the amplitude of aggregation as a parameter *K*, defined in Eq. (24), exceeds *j*_11_/*l*. We have checked this transition by using the exact Lyapunov functional, simplified for radially symmetric solutions [Eq. (46)]. The system converges to two different states depending on in which direction the transition point is approached. At the transition point, however, *W* is independent of the amplitude of aggregation and a continuous spectrum of infinitely many states exists between the two states with exactly the same value of *W*. The transition is thus continuous.

Our numerical calculation furthermore shows that Eq. (46) has multiple local minima (Figs 3 and 4). It is an open question if the existence of multiple local minima in *W* is due to the fact that we have restricted ourselves to radially symmetric solutions. That is, if we relaxed the symmetry requirement, some of the local minima could be connected to others via non-symmetric states. For example, the annular structure in Fig. 3(a) has relatively high *W* than other minima, and it is likely to collapse into another state in the presence of non-symmetric perturbations. At the same time, the extended parameter space could well introduce far more metastable states in the absence of the radial symmetry: Reference 28 shows us one of such states obtained with the finite-element method. To check those possibilities, we are currently working with the full normal-mode expression of *W* without the radial symmetry.

## Method

In minimizing a partial sum of *W* from *m* = 0 to $m=\hat{m}$ [Eq. (46)] numerically, we treat the total mass *M* [Eq. (4)] and temperature *T* as input parameters. The initial state is defined by a set of variables, *G*_00_ ≡ *c*_const_ = *M*/(*πl* ^2^) and ${E}_{01}={E}_{02}=\cdots ={G}_{01}={G}_{02}=\cdots =0$, from which *W* is computed. Note that *E*_00_ ≡ *ρ*_const_ = *M*/(*πl* ^2^) is a constant that will not be updated throughout the minimization procedure. We generate a neighbouring state in the following way: We first choose a mode $m\in \mathrm{[0},\ldots ,\hat{m}]$. If *m* > 0, we add two independent random numbers *r*_*E*_ and *r*_*G*_, each of which is taken from [−0.1, 0.1), to the corresponding amplitudes *E*_0*m*_ and *G*_0*m*_, respectively. If *m* = 0, on the other hand, only *G*_00_ will be updated by *r*_*G*_ ∈ [−0.1, 0.1) because *E*_00_ = *ρ*_const_ should remain constant. From this neighbouring state, we can calculate the Lyapunov functional, and let us denote its value *W*′. We basically employ the standard Metropolis algorithm to determine whether to accept the move to this neighbouring state: We first check if the move satisfies *W*′ ≤ *W*. Otherwise, we draw a random number from [0, 1) and check if it is smaller than exp[(*W* − *W*′)/*T*]. If either of those two conditions is met, we proceed to check if the move leaves both *ρ* and *c* non-negative everywhere inside the disc by dividing the region into a sufficiently fine mesh compared to the variations of the highest mode with $\hat{m}$. In short, we carry out the move only if it is accepted by the Metropolis algorithm without violating the non-negativity. One Monte Carlo step consists of $(\hat{m}+\mathrm{1)}$ such attempts to move to neighbouring states.

We test the algorithm by running it at *T* = 0 to obtain the expected results such as in the inset of Fig. 4(a). To find a better minimum of *W*, we choose an annealing schedule as *T* = 10 × (1.2)^−*n*^ with *n* = 0, 1, …, 50, and take 1.5 × 10^4^ Monte Carlo steps at each *T* (Figs 3 and 4). We also note that we have added calculations with *T* = 0 at the end of this annealing schedule for clarity in Fig. 2.
